# Gene Expression Commons: An Open Platform for Absolute Gene Expression Profiling

**DOI:** 10.1371/journal.pone.0040321

**Published:** 2012-07-18

**Authors:** Jun Seita, Debashis Sahoo, Derrick J. Rossi, Deepta Bhattacharya, Thomas Serwold, Matthew A. Inlay, Lauren I. R. Ehrlich, John W. Fathman, David L. Dill, Irving L. Weissman

**Affiliations:** 1 Institute for Stem Cell Biology and Regenerative Medicine, Stanford University School of Medicine, Stanford, California, United States of America; 2 Department of Computer Science, Stanford University, Stanford, California, United States of America; University of Jaén, Spain

## Abstract

Gene expression profiling using microarrays has been limited to comparisons of gene expression between small numbers of samples within individual experiments. However, the unknown and variable sensitivities of each probeset have rendered the absolute expression of any given gene nearly impossible to estimate. We have overcome this limitation by using a very large number (>10,000) of varied microarray data as a common reference, so that statistical attributes of each probeset, such as the dynamic range and threshold between low and high expression, can be reliably discovered through meta-analysis. This strategy is implemented in a web-based platform named “Gene Expression Commons” (https://gexc.stanford.edu/) which contains data of 39 distinct highly purified mouse hematopoietic stem/progenitor/differentiated cell populations covering almost the entire hematopoietic system. Since the Gene Expression Commons is designed as an open platform, investigators can explore the expression level of any gene, search by expression patterns of interest, submit their own microarray data, and design their own working models representing biological relationship among samples.

## Introduction

**Figure 1:**
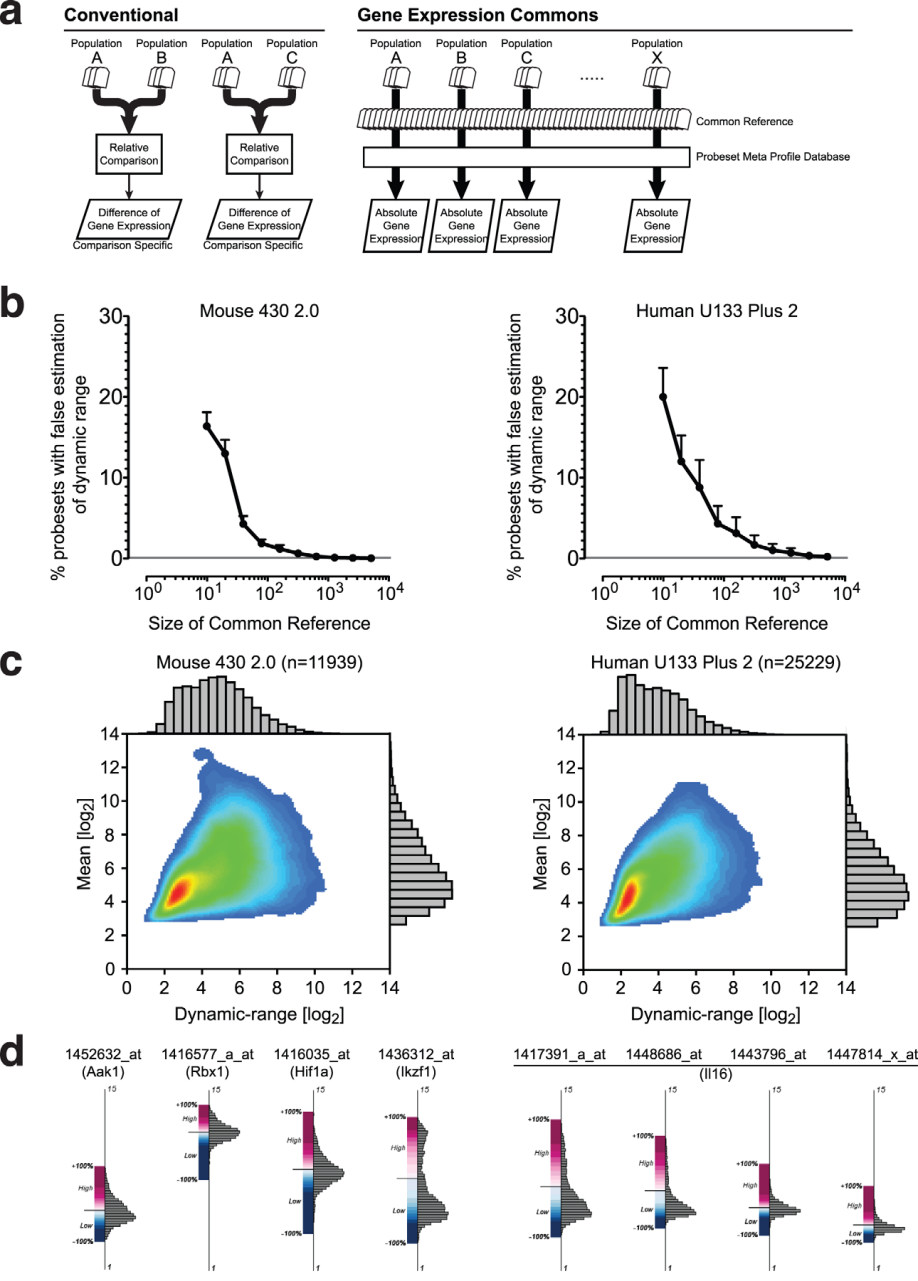
Absolute gene expression profiling with a large-scale common reference and a probeset meta profile database. (A) Relative vs. absolute gene expression profiling. Conventional methods compare differences in gene expression between samples within an individual experiment, and result in relative values only (left). In Gene Expression Commons, raw microarray data is individually normalized against a large-scale common reference, then mapped onto the probeset meta profile. This strategy enables profiling of absolute expression levels of all genes on the microarray (right). (B) Relationship between the size of the common reference and the accuracy of the probeset dynamic range estimation. The result of one out of five experiments is shown. The Y-axis represents % probesets with false estimation of dynamic range in mean ± S.E.M (n = 10). (C) The dynamic range versus the mean of each probeset in Affymetrix Mouse 430 2.0 (n = 11,939) (left) and Affymetrix Human U133 Plus 2 (n = 25,229) (right) presented by a density plot and histograms. (D) Graphical representation of probeset meta profile. The Y-axis represents expression intensity without units in log2 scale. The distribution of expression levels is displayed by a histogram (right side of the axis). The high/low threshold computed is shown by a solid bar, and the distribution of percentiles in either the high or low expression range is indicated by a gradation of color, displayed as highest (+100%) in dark red, threshold (0%) in white, lowest (−100%) in dark blue (left side of the axis). Four diverse distributions of probesets for four different genes (Aak1, Rbx1, Hif1a and Ikzf1) (left), and diverse distribution of four probesets of one gene (Il16) (right) are shown.

Gene expression microarray technology has allowed a global measurements of gene expression status in diverse cells and tissues across species [1]. Technological advances enable integration of thousands of probesets on a single chip, providing mRNA levels to be measured for almost all known protein-coding gene in the genome. However, since each probeset intrinsically has a different efficiency of hybridization due to differences in target sequences, current methodologies of array-based global gene expression analysis are limited to profiling *relative* differences in gene expression among samples rather than *absolute* gene expression analyses of a particular sample [1] (Figure 1A left). The output of differentially regulated genes is unique to the combination of samples compared, and the relative differences are frequently misinterpreted because there is no assurance that every probeset has the same dynamic range of gene expression values. For instance, the biological interpretation of 2-fold changes (1 log2 shift) for a probeset that has small dynamic range compared to a probeset that has large dynamic range will be different. Moreover, genes whose expression level does not vary significantly between samples within a given study are frequently ignored regardless of their expression intensity. These limitations have created a bottleneck for generalizing results of gene expression microarray assays across experiments and across laboratories.

To overcome these limitations, we hypothesized that a very large number of publicly available microarray data from a particular microarray platform could be used as a common reference that might be enable empirical estimation of the absolute expression level of a given gene. If the common reference is large enough, meta-analysis could be applied to the common reference to compute the distribution of data, dynamic-range, and a threshold to distinguish high expression from low expression for each probeset. Using such an approach, we now demonstrate that absolute gene expression profiling can be achieved by mapping sample data against a common reference obtained by meta-analysis (Figure 1A right).

## Results

### Common Reference and Probeset Meta-Profile Database

In our initial experiment, we determined the size of the common reference required to perform meta-analysis with sufficient statistical power. We focused on Affymetrix microarrays because of the abundance of publicly available data. We downloaded 11,939 Affymetrix Mouse Genome 430 2.0 and 25,299 Affymetrix Human Genome U133 Plus 2.0 gene expression microarray data from the NCBI Gene Expression Omnibus (GEO) public repository [2]. From the pool of microarray data downloaded, we randomly selected 10, 20, 40, 80, 160, 320, 640, 1280, 2560, and 5120 data as a hypothetical common reference. Each common reference sample was normalized using the RMA algorithm [3], and for each probeset, the dynamic range was calculated as the difference between the lowest and the highest expression values among the data. Next, one additional microarray data was randomly chosen from the pool as the “actual sample” and was normalized with each hypothetical common reference. To test if adding the “actual sample” changed the computed dynamic ranges of probesets significantly, we analyzed the number of probesets for which the expression intensity of the “actual sample” falls outside the dynamic range calculated from the hypothetical common reference. This number decreased exponentially as the size of hypothetical common reference increased (Figure 1B). As shown in Figure 1B, the dynamic range becomes stable as the size of the hypothetical common reference approaches 2560, and the probabilities of false estimation of the probeset dynamic range are less than 0.1% for mouse, and 0.5% for human, respectively. We repeated this experiment five times and observed consistent trends (Table 1 and 2). This result indicates that if large numbers of microarray data are pooled and normalized together, we can estimate the dynamic range of each probeset with a high degree of confidence, and can use those pooled data as a “Common Reference”. To maximize confidence, we used all 11,939 Affymetrix mouse 430 2.0 microarray data and 25,229 Affymetrix human U133 Plus 2.0 microarray data for the first version of the common reference.

**Table 1:** Size of universal reference and probeset dynamic-range estimation Affymetrix Mouse 430 2.0 (45101 probesets).

False positive on probeset dynamic-range estimation (mean±s.e.m.) [%]										
	Size of common reference (number of microarrray data)									
Exp	10	20	40	80	160	320	640	1280	2560	5120
#1 (n = 10)	16.38±1.72	12.99±1.69	4.28±0.98	1.88±0.48	1.19±0.48	0.63±0.27	0.25±0.11	0.11±0.04	0.07±0.03	0.04±0.02
#2 (n = 10)	15.10±1.83	10.67±2.30	3.95±0.91	2.26±0.72	1.00±0.35	0.59±0.25	0.27±0.09	0.14±0.07	0.06±0.02	0.03±0.01
#3 (n = 10)	17.68±2.16	7.50±1.16	4.51±1.02	2.52±0.55	1.18±0.44	0.54±0.19	0.21±0.06	0.12±0.05	0.05±0.02	0.04±0.02
#4 (n = 10)	18.37±2.85	10.88±1.49	5.25±1.11	2.59±0.85	1.05±0.30	0.48±0.15	0.32±0.15	0.13±0.06	0.07±0.03	0.03±0.01
#5 (n = 10)	19.15±2.26	7.86±1.09	4.63±1.10	2.19±0.54	1.27±0.56	0.53±0.17	0.25±0.09	0.13±0.06	0.05±0.02	0.03±0.01
Ave	17.34±0.72	9.98±1.02	4.52±0.22	2.29±0.13	1.14±0.05	0.55±0.03	0.26±0.02	0.13±0.00	0.06±0.00	0.03±0.00

**Table 2:** Size of universal reference and probeset dynamic-range estimation Affymetrix Human U133 Plus 2 (54677 probesets).

False positive on probeset dynamic-range estimation (mean±s.e.m.) [%]										
	Size of common reference (number of microarrray data)									
Exp	10	20	40	80	160	320	640	1280	2560	5120
#1 (n = 10)	20.01±3.58	12.01±3.23	8.78±3.42	4.32±2.18	3.14±1.97	1.69±1.18	1.03±0.78	0.71±0.57	0.34±0.28	0.23±0.20
#2 (n = 10)	24.18±3.89	13.85±3.85	8.28±3.20	4.73±2.35	2.63±1.52	1.44±0.98	0.99±0.71	0.72±0.61	0.46±0.40	0.30±0.28
#3 (n = 10)	22.28±4.00	10.00±2.49	6.91±2.44	3.56±1.86	2.67±1.49	1.56±1.10	0.98±0.73	0.60±0.47	0.42±0.36	0.32±0.29
#4 (n = 10)	20.89±3.77	12.32±3.04	8.70±3.24	5.31±2.66	2.98±1.90	1.92±1.36	1.23±0.96	0.66±0.53	0.53±0.47	0.20±0.18
#5 (n = 10)	19.11±3.77	12.96±3.68	5.78±2.22	5.75±2.98	3.24±1.98	1.73±1.20	1.13±0.87	0.53±0.43	0.58±0.52	0.27±0.24
Ave	21.30±0.89	12.23±0.64	7.69±0.58	4.73±0.38	2.93±0.12	1.67±0.08	1.07±0.05	0.65±0.04	0.46±0.04	0.27±0.02

Since the common reference contains a large number of data points for each probeset, various attributes besides the dynamic-range can be calculated with statistical reliability. Figure 1C displays the scatter plot of the dynamic range versus the mean of each probeset obtained from Affymetrix mouse 430 2.0 microarrays (n = 11,939) and Affymetrix human U133 Plus 2.0 microarrays (n = 25,229). The dynamic range varies widely from 1 to 10 in log2 scale in both platforms. This variation clearly indicates the limitations of conventional selection criteria based on just the relative fold-change between samples within a single experiment. The biological context of a 2-fold increase in a probeset that has a small dynamic range is likely to be more significant than a 2-fold increase in a probeset in which the highest level of expression is much more than a 2-fold change. Conversely, one cannot expect to find a 10-fold change in expression of a gene with a small dynamic range.

Another important finding is that there is wide variation in the mean values of probesets with the same dynamic range. Two different possibilities may explain this phenomenon. First, non-specific hybridization signals are significantly different for different probesets. Second, there are a large number of “housekeeping” genes that are highly expressed irrespective of cell type. On the microarray, both of these two types of probesets are likely present, but our approach does not distinguish between them.

Because of the large sample size in our common reference, the distribution of gene expression values for each probeset is extremely stable. Therefore, the inclusion of additional data in the future will likely not affect this distribution, as shown in Figure 1B. Further, we can infer that for each probeset large numbers of representative low and high gene expression values are available. Thus, a threshold value which distinguishes high expression from low expression can be estimated (high/low threshold). To achieve this, we used the StepMiner algorithm originally developed to fit step functions [4]. For each probeset, the expression intensities were sorted from low to high, and a step function fitted to the sorted expression intensities that minimizes the square error between the original and the fitted values. This enabled us to identify a threshold for high versus low expression. Each individual expression value from the common reference for a given probeset was assigned a percentile rank. For each probeset, the meta database provides the high/low threshold, minimum, maximum.

A graphical presentation of the meta profiles for eight representative probesets appears in Figure 1D. The Y-axis represents expression intensity. On the right side of the axis, the distribution of expression intensities of the data used to generate the common reference is displayed in a histogram. On the left side of the axis, the high/low threshold is shown by a solid bar, and the distribution of percentiles in either the high or low expression range is displayed by a gradation of color. The probeset for Aak1 (1452632_at) and the probeset for Rbx1 (1416577_a_at) have almost equivalent dynamic ranges, but their distributions of expression intensities are strikingly different. The probeset for Hif1a (1416035_at) has a uni-modal distribution, whereas the probeset for Ikzf1 (1436312_at) has a bimodal distribution. The Affymetrix Mouse 430 2.0 microarray contains 4 probesets for Il16, and each probeset has a distinct dynamic-range and shape of distribution (Figure 1D, right).

### Gene Expression Commons

We developed a computer program to normalize microarray data of interest against the common reference. As a result of this processing, the expression intensities and expression percentiles are obtained for all probesets, and the stability of the results is greater than 99.9%. Thus, absolute gene expression profiling can be achieved. We integrated these strategies into an intuitive web interface named “Gene Expression Commons” (https://gexc.stanford.edu).

**Figure 2:**
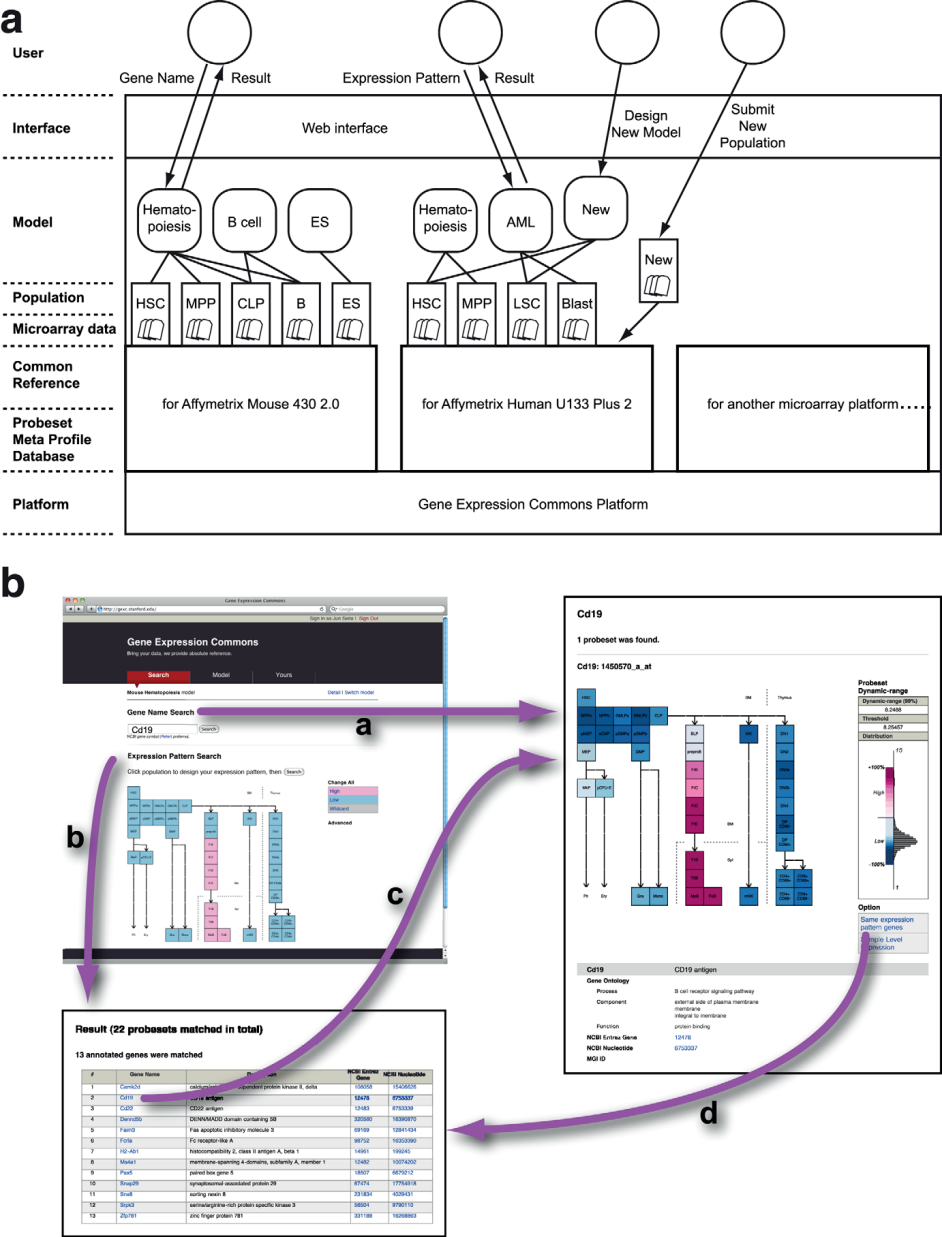
Structure and Workflow of Gene Expression Commons. (A) Functional layers of Gene Expression Commons system. Users can select a Model of interest, and search for absolute gene expression through an intuitive web interface. A Model is a searchable category representing a biological context and displaying relationships among Populations. A Population contains several microarray data, which are biological replicates. Users can submit their own Populations, and design Models with a privacy control feature. (B) Seamless search flow at Gene Expression Commons. Gene Name Search provides absolute gene expression of a particular gene (path a). Expression Pattern Search provides a list of genes with expression patterns matching the expression pattern of interest designed by user (path b). From the list of genes, absolute expression of a particular gene is displayed with one click. From the absolute gene expression of a particular gene, the user can obtain a list of genes with the same expression pattern (path d).

Figure 2A shows each functional layer of Gene Expression Commons. Users can search for absolute gene expression with probeset meta profile information using a web interface.

“Model” is a searchable category that represents biological context and relationships among “Populations” displayed in 2-dimensions (e.g. Mouse Hematopoiesis Model, described later). “Population” is the most essential unit of data in Gene Expression Commons, which contains several microarray data that are biological replicates. Microarray data are normalized individually with the common reference. Then, the normalized values are averaged. These averaged values are then mapped onto the probeset meta-profile in order to obtain the population’s percentile of expression for each probeset.

The system is designed to enable updating of the common reference to improve the breadth of available samples. When the size of publically-available data of Affymetrix mouse 430 2.0 microarray or Affymetrix human U133 Plus 2.0 microarray has increased significantly, e.g. doubled from current size, we will introduce a new version of the common reference. Also, other microarray platforms will be added to the system when the numbers of publicly available data reaches a point at which generation of a stable common reference is possible.

### Mouse and Human Hematopoiesis Models

Hematopoietic stem cells generate more than 10 distinct functional cell types every second through multiple intermediate progenitor stages. Hematopoietic stem cells and many of their downstream progeny can be readily isolated and molecularly characterized. To establish a gene expression map of hematopoiesis, we highly purified 39 defined hematopoietic populations for which we have established putative differentiation pathways (Table 3) from bone marrow, spleen, and thymus utilizing 12-color digital FACS, and generated gene expression data for each on the Affymetrix Mouse Genome 430 2.0 microarray platform (GSE34723) [5], [6]. The data of each population were incorporated into Gene Expression Commons, and a “Mouse Hematopoiesis” model was generated (Figure 2B). On the Mouse Hematopoiesis model, users can search and observe the absolute gene expression profile of any gene on Affymetrix Mouse Genome 430 2.0 microarray platform simply by searching for the NCBI gene symbol, gene name, keyword, or probeset ID. For instance, the gene expression profile of CD19 is shown in Figure 2B (path a). On the results page, the right column displays the probeset meta profile including its dynamic range, threshold level, histogram of data distribution, and calculated low/high expression level represented by a color-coded heatmap. The left column depicts the expression level of the gene searched in a heatmap. Red represents high expression, white represents threshold level expression, and blue represents low expression. Because of the size of the common reference data, the expression levels for each population are stable. Thus, adding another populations to the “Mouse Hematopoiesis” model in the future will not alter the expression levels of existing populations. This feature is very important, as discovery of new cell populations will undoubtedly occur.

**Table 3:** Populations in Mouse Hematopoiesis Model.

#	Name	Definition	Organ	Strain	Age [weeks]	Sex	Replicate	Reference
1	HSC	CD34− Flk2− Sca-1+ c-Kit+ Il7ra− Lin(CD4, CD8, B220, Ter119, Mac1, Gr1)-	BM	C57Bl/6	12	M	4	
2	MPPa	CD34+ Slamf1+ Tie2+ Vcam1+ Sca-1+ c-Kit+ Il7ra− Lin(CD4, CD8, B220, Ter119, Mac1, Gr1) −	BM	C57Bl/6	12	M	3	
3	MPPb	CD34+ Slamf1− Tie2+ Vcam1+ Sca-1+ c-Kit+ Il7ra− Lin(CD4, CD8, B220, Ter119, Mac1, Gr1) −	BM	C57Bl/6	12	M	3	
4	GMLPa	CD34+ Slamf1− Tie2− Vcam1+ Sca-1+ c-Kit+ Il7ra− Lin(CD4, CD8, B220, Ter119, Mac1, Gr1) −	BM	C57Bl/6	12	M	3	
5	GMLPb	CD34+ Slamf1− Tie2− Vcam1− Sca-1+ c-Kit+ Il7ra− Lin(CD4, CD8, B220, Ter119, Mac1, Gr1) −	BM	C57Bl/6	12	M	3	
6	pMEP	CD34+ FcgRII/IIIlow Slamf1+ Tie2+ Vcam1+ Sca-1− c-Kit+ Il7ra− Lin(CD4, CD8, B220, Ter119,Mac1, Gr1) −	BM	C57Bl/6	12	M	3	
7	sCMP	CD34+ FcgRII/IIIlow Slamf1− Tie2+ Vcam1+ Sca-1− c-Kit+ Il7ra− Lin(CD4, CD8, B220, Ter119,Mac1, Gr1) −	BM	C57Bl/6	12	M	3	
8	pGMPa	CD34+ FcgRII/IIIlow Slamf1− Tie2− Vcam1+ Sca-1− c-Kit+ Il7ra- Lin(CD4, CD8, B220, Ter119, Mac1, Gr1) −	BM	C57Bl/6	12	M	3	
9	pGMPb	CD34+ FcgRII/IIIlow Slamf1− Tie2− Vcam1− Sca-1− c-Kit+ Il7ra− Lin(CD4, CD8, B220, Ter119,Mac1, Gr1) −	BM	C57Bl/6	12	M	3	
10	MEP	CD34− FcgRII/III− Sca-1− c-Kit+ Il7ra− Lin(CD4, CD8, B220, Ter119, Mac1, Gr1) −	BM	C57Bl/6	12	M	3	
11	MkP	Slamf1+ CD41+ Sca-1− c-Kit+ Lin(CD4, CD8, B220, Ter119, Mac1, Gr1) −	BM	C57Bl/6	8∼12	M/F	3	[20]
12	preCFU-E	Slamf1+ CD105+ CD41− FcgRII/III- Sca-1− c-Kit+ Lin(CD4, CD8, B220, Ter119, Mac1, Gr1) −	BM	C57Bl/6	8∼12	M/F	3	[20]
13	GMP	CD34+ FcgRII/III+ Sca-1− c-Kit+ Il7ra− Lin(CD4, CD8, B220, Ter119, Mac1, Gr1) −	BM	C57Bl/6	12	M	3	[5]
14	Gra	Gr1+ Mac1+ CD105− Sca-1− c-Kit- Il7ra- Lin(CD4, CD8, B220, Ter119)−	BM	C57Bl/6	12	M	2	
15	Mono	Gr1− Mac1+ CD105+ Sca-1− c-Kit− Il7ra- Lin(CD4, CD8, B220, Ter119) −	BM	C57Bl/6	12	M	2	
16	CLP (ALP)	Lin (Mac1, Gr1, Ter119, CD3, CD19) − CD11c− B220− CD27+ ckitint Flk2+ IL7Ra+ Ly6d−	BM	C57Bl/6	8–12	M	3	[6]
17	BLP	Lin (Mac1, Gr1, Ter119, CD3, CD19) − CD11c− B220− CD27+ ckitint Flk2+ IL7Ra+ Ly6d+	BM	C57Bl/6	8–12	M	3	[6]
18	pre-pro-B	Lin (Mac1, Gr1, Ter119, CD3, CD19) − CD11c− B220+ CD27+ ckitint Flk2+ IL7Ra+ Ly6d+	BM	C57Bl/6	8–12	M	4	[6]
19	FrB2	Lin (Mac1 Gr1 Ter) − B220+ CD11c− CD19+ CD27− IgD− IgM− IL7Ra+6C3− CD43+	BM	C57Bl/6	9–12	M	2	
20	FrC	Lin (Mac1 Gr1 Ter) − B220+ CD11c− CD19+ CD27− IgD− IgM− IL7Ra+6C3+ CD43+	BM	C57Bl/6	9–12	M	2	
21	FrD	Lin (Mac1 Gr1 Ter) − B220+ CD11c− CD19+ CD27− IgD− IgM− IL7Ra− 6C3− CD43−	BM	C57Bl/6	9–12	M	2	
22	FrE	Lin (Mac1 Gr1 Ter) − B220+ CD11c− CD19+ CD27− IgD− IgM+ IL7Ra− 6C3− CD43−	BM	C57Bl/6	9–12	M	2	
23	T1	CD19+ IgMlo IgD− CD23− AA4.1+	Spleen	C57Bl/6	9–12	M	2	
24	T2	CD19+ IgMlo IgD− CD23+ AA4.1+	Spleen	C57Bl/6	9–12	M	2	
25	Mz B	CD19+ IgM+ CD21+ CD23lo AA4.1−	Spleen	C57Bl/6	9–12	M	2	
26	Fo B	CD19+ IgMlo IgD+ CD21lo CD23+ AA4.1−	Spleen	C57Bl/6	9–12	M	2	
27	iNK	DX5− CD122+ Mac1− CD19− NK1.1+ CD27+ CD11c− CD3− Ly6D−	BM	C57Bl/6	12	M	2	
28	mNK	DX5+ CD122+ Mac1+ CD19− NK1.1+ CD27+ CD3− Ly6D−	Spleen	C57Bl/6	12	M	2	
29	DN1	Mac-1− NK1.1− CD11c− gamma-delta- B220− CD4− CD8− cKit+ CD44+ CD25−	Thy	C57Bl/6	5	M/F	3	[5]
30	DN2	Mac-1− NK1.1− CD11c− gamma-delta- B220− CD4− CD8− cKit+ CD44+ CD25+	Thy	C57Bl/6	5	M/F	3	[5]
31	DN3a	Mac-1− NK1.1− CD11c− gamma-delta- B220− CD4− CD8− thy1.1+ CD3− CD44−CD25+ FSC-A lo CD27lo	Thy	C57Bl/6	5	M	3	[5]
32	DN3b	Mac-1− NK1.1− CD11c− gamma-delta- B220− CD4− CD8− thy1.1+ CD3− CD44− CD25+ FSC-A hi CD27hi	Thy	C57Bl/6	5	M	3	
33	DN4	Mac-1− NK1.1− CD11c− gamma-delta- B220− CD4− CD8− thy1.1+ CD3− CD44− CD25−	Thy	C57Bl/6	5	M	3	
34	DP CD69-	Mac-1− NK1.1− CD11c− gamma-delta- B220−CD4+CD8+CD69−	Thy	C57Bl/6	5	M	3	
35	DP CD69+	Mac-1− NK1.1− CD11c− gamma-delta- B220−CD4+CD8+CD69+	Thy	C57Bl/6	5	M	3	
36	CD4SP CD69+	Mac-1− NK1.1− CD11c− gamma-delta- B220−CD4+CD8−CD3+CD69+	Thy	C57Bl/6	5	M	3	
37	CD4SP CD69−	Mac-1− NK1.1− CD11c− gamma-delta- B220−CD4+CD8−CD3+CD69−	Thy	C57Bl/6	5	M	3	
38	CD8SP CD69+	Mac-1− NK1.1− CD11c− gamma-delta- B220−CD4−CD8+CD3+CD69+	Thy	C57Bl/6	5	M	3	
39	CD8SP CD69−	Mac-1− NK1.1− CD11c− gamma-delta- B220−CD4−CD8+CD3+CD69−	Thy	C57Bl/6	5	M	3	

BM: bone marrow; Thy: thymus.

If a microarray platform has multiple probesets for a particular gene, the Gene Expression Commons sorts the results based on the dynamic range of each probeset. To eliminate potential outliers, the highest 0.5% and lowest 0.5% of data points are ignored in this dynamic-range calculation. Probesets are sorted from greatest to least dynamic range, since the probeset with the largest dynamic range is likely to be the most informative. However, a probeset that has wide and single-modal distribution could be probeset with greater noise.

For each probeset, the Gene Expression Commons displays not only the dynamic range, but also the threshold value, which distinguishes high from low expression. This enables searches based upon particular expression patterns. For instance, genes expressed only in the B-cell development pathway can be searched by a few clicks (Figure 2B, path b). A list of genes with an expression pattern matching the inquiry is displayed, and the user can then investigate the detailed gene expression profile with one more click (Figure 2B, path c).

**Figure 3:**
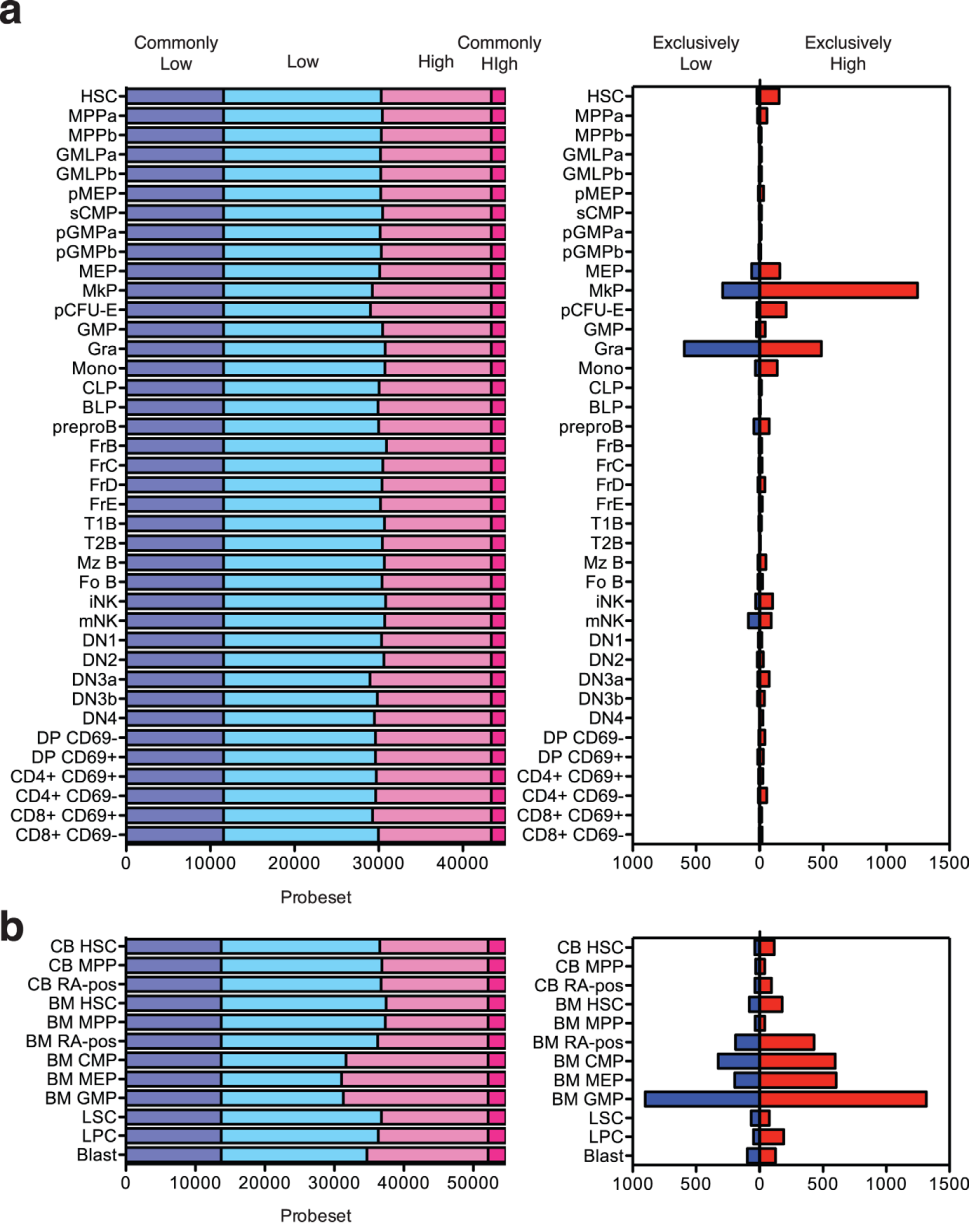
Gene expression pattern profiling of mouse and human hematopoiesis. Number of genes expressed at very low levels (dark blue), low levels (light blue), high levels (light red) and very high levels (dark red) in either the Mouse Hematopoiesis Model (A, left) or Human Hematooiesis Model (B, left). Number of genes expressed at high (red) and low (blue) levels in a specific population in Mouse Hematopoiesis Model (A, right) and Human Hematopoiesis Model (B, right). A list of genes matching each criterion can be obtained with a few clicks on Gene Expression Commons (https://gexc.stanford.edu/).

Using this powerful feature, Expression Pattern Search, we can profile the kinetics of gene expression within the model. For instance, genes expressed at a high level in all of the populations in the Mouse Hematopoiesis model can be searched by one click. In the Mouse Hematopoiesis model, 1722 probesets are always high, and 11,569 probesets are consistently low. Other genes dynamically change expression among the populations (Figure 3A, left). This type of profiling cannot be achieved by conventional relative comparisons of small numbers of samples.

Furthermore, genes highly expressed in a specific population can be identified (Figure 3A, right). Interestingly, very few genes specific for a particular hematopoietic population (either high or low) are detected in the Mouse Hematopoiesis model. This result suggests that gene expression programs change gradually throughout hematopoietic differentiation. Lists of genes and further details can be obtained at Gene Expression Commons (https://gexc.stanford.edu/).

Gene expression microarray data of publicly available human hematopoietic populations [7], [8] are also available in the Gene Expression Commons. This Human Hematopoiesis model contains HSC, MPP, and RA-positive progenitor population from cord blood; HSC, MPP, RA-positive progenitor population, CMP, MEP, and GMP from healthy adult bone marrow; and LSC, LPC, and Blast populations from AML patients.

### Gene Expression Commons as an Open Platform

Gene Expression Commons is not only a search engine for existing gene expression data, but also a universal open platform to profile absolute gene expression of any microarray data. Users can submit gene expression microarray data files of their own, or published microarray data downloaded from public repositories e.g. GEO. Users can also design their own models by combining “Populations” to represent biological context of interest. Users can control the privacy level of submitted data and models, and share private models using group function provided by web platform. Through this workflow, users can find key genes and expression patterns by absolute gene expression profiling to identify candidates relevant to their biological question of interest. Once a project is published, the population and model used will be publicly available at the Gene Expression Commons.

## Discussion

We described a strategy for absolute gene expression profiling based on meta-analysis of large-scale microarray data, and introduced the Gene Expression Commons system as a comprehensive discovery platform. The common reference and probeset meta database provide significant advantages over conventional relative gene profiling. For each microarray, gene expression can be measured relative to the common reference that provides absolute gene expression values for comparison between many microarray experiments.

The concept of using a common reference to normalize large amounts of Affymetrix data was first proposed and developed by Katz et al. [9] in 2006. Katz et al. used 1,614 diverse biological samples comprised of 251 tissue and pathological categories as the common reference. In 2007, Day et al., described the Celsius system in which by a selected ‘quantification pool’ of 50 heterogeneous mixture of samples is held constant for all quantification events [10]. However, as we shown by computational simulation in Figure 1b, this size of data pool is not large enough to establish stable common reference. In our system we used almost all of the publicly available gene expression data (>10,000 arrays) to enhance the stability of the common reference. However, the drawback of this large-scale common reference strategy is the computational cost of the normalization process. Each time a new microarray is submitted, the data is re-normalized against all of the microarrays in large-scale common reference, which takes several hours on a dedicated server. Recently, McCall et al. expanded the work of Katz et al. and develop a new strategy called frozen RMA (fRMA) [11]. fRMA first computes probe-specific parameters required for normalization from large-scale publicly available microarray data, then normalizes each additional microarray using those pre-computed values. Therefore, the fRMA method has a significant potential to improve the processing speed of normalization in Gene Expression Commons.

Oncomine is a database of cancer microarrays and provides a platform for differential expression analyses comparing most major types of cancer with respective normal tissues as well as a variety of cancer subtypes [12]. In databases like Oncomine, Flymine [13], Ingenuity, EMAAS [14], MiMiR [15] and many others around the world, microarray data can be imported, queried and visualized for a selected gene across all analyses or for multiple genes in a selected analysis. However, none of these databases provide an analysis platform for absolute gene expression profiling. In 2007, Zilliox et at introduced a method to compute thresholds that distinguish expressed from unexpressed genes, as part of a system to define tissue-specific ‘gene expression bar codes’, using 1092 manually curated human samples and 236 mouse samples [16]. They added a web interface in 2011 to obtain a bar code for a particular sample uploaded by user (http://rafalab.jhsph.edu/barcode/index.php) [17]. In this method, the authors used the smallest mode, defined as local maximum of the estimated density distribution, and standard deviation estimated from expression estimates to the left side of the smallest mode. By contrast, Gene Expression Commons uses random samples from a large pool of microarray data as common reference, and sets a threshold to divide the expression of each gene into “low” and “high” values (instead of “present” and “absent”). This threshold is computed by sorting the expression values from low to high, then using the StepMiner algorithm to fit a step function to the data. Low and high values may be more appropriate for finding signature genes that differentiate cell types, since genes are often expressed at a low level in many cell types, but at dramatically higher levels in a small number of cell types of interest. We would encourage investigators to experiment with web interfaces for both systems to find out which method is most appropriate for their purposes.

Since hematopoiesis has been one of the most studied tissue stem cell based systems, numerous efforts have been invested in microarray analysis of hematopoietic cells, especially hematopoietic stem cells (Table 4). However, each study used a different protocol for the purification of HSCs, and used different cell populations as the counterpart to obtain ‘differentially regulated genes’. Thus each result is project-specific and is difficult to generalize.

**Table 4:** Microarray gene expression profilings tageting HSC based on relative comparison.

Year	Group	Platform	HSC	Compared to	# of probes	# of genes	Ref
2002	Hood	House made	Rhlow KLS	Rhhigh KLS		5000	[21]
2002	Lemischka	Affymetrix MU-U74-2 A∼C	Rhlow KLS	Variou type of cells	36000	6000+18000 EST	[22]
2003	Akashi	Affymetrix MU-U74-2 A∼C	Thy1.1low Rhlow KLS	MPP, CMP, CLP	36000	6000+18000 EST	[23]
2003	Weissman	Clontech Atlas Mouse cDNA array	Thy1.1low KLS	CMP, CLP, GMP, MEP, ProT, ProB		1200	[24]
2005	Weissman	Stanford Microarray Facility 42 kmouse cDNA array	Thy1.1low Flk2− KLS	Thy1.1low Flk2+ KLS, Thy1.1−Flk2+ KLS	42000		[25]
2005	Weissman	Affymetrix Mouse 430 2.0	CD34− Flk2− KLS (young)	CD34− Flk2− KLS (old)	45000	34000	[26]
2006	Goodell	Affymetrix Mouse U74A	SP Sca1+ Gr1−	CD8+ T cell	45000	34000	[27]
2007	Goodell	Affymetrix Mouse 430 2.0	SP KLS	Erythrocyte, Granulocyte,Native T cell, Activated T cell,Activated B cell, Monocyte,NK cell	45000	34000	[28]

KLS: c-Kit+ Lin− Sca-1+; MPP: multipotent progenitor; CMP: common myeloid progenitor; CLP: common lymphoid progenitor; GMP: granulocyte/macrophage progenitor; MEP: megakaryocyte/erythrocyte progenitor; Pro T: progenitor T cell, Pro B: progenitor B cell.

Recently, more comprehensive approaches to profile gene expression of hematopoietic systems have been introduced. BloodExpress collected published microarray data of 37 distinct mouse hematopoietic populations *ex vivo* or after culture, as conducted by 15 different projects/laboratories [18]. BloodExpress processed each microarray data by the MAS5 method which does not consider differences in the dynamic ranges of probesets, and each gene was classified into binary Present or Absent states. In terms of data processing strategy, BloodExpress’ binarization by MAS5 was a practical first-pass categorization of gene expression. On the other hand, the data integrated into BloodExpress were highly heterogeneous, and the classification of all genes into “present” or “absent” categories is overly simplistic.

In 2008, Heng and Painter proposed an aspiring project named ‘Immgen Project’ to establish a complete ‘road map’ of gene-expression and regulatory networks in all immune cells [19]. This project is aiming to generate microarray data of over 200 immune cell types by a highly standardized protocol. However, they do not provide absolute gene expression because their arrays are not compared with arrays from other tissues.

To overcome those limitations, we sorted and profiled 39 mouse hematopoietic populations using very strict cell surface criteria, and the most modern sorting strategies. All these data have been loaded onto the Gene Expression Commons and will be made available to the public. Moreover, because of the advantage of the common reference strategy, incorporating additional data of new populations in future will not detectably change the gene expression readout of existing populations. Thus, it is our belief that the Gene Expression Commons will serve as a common platform for absolute profiling of gene expression in the hematopoietic system.

The Gene Expression Commons has many other potential uses. For example, one can enter the name of a gene, and rapidly determine the quantitative expression of that gene in each cell type. Alternatively, one can query any cell type within a model to obtain a list of genes expressed exclusively in that cell type, or concomitantly with a defined subset of other cell types. This could be done in mice, where mutant and lineage tracing strains exist to identify candidate genes that may be important in cellular differentiation. Another possible use is for pharmacology, where the expression of drug targets and potential toxicities to hematopoietic stem and progenitor cells can be predicted.

Here we demonstrate that absolute gene expression profiling can be achieved by establishing large-common reference data and meta-analysis. This strategy advances gene expression analysis beyond conventional profiling with small numbers of samples. Additionally, this strategy can be applicable to other platforms for high-throughput assays including exon arrays, microRNA arrays, or DNA methylation arrays. The strategy is implemented into a web-based open platform termed “Gene Expression Commons” (https://gexc.stanford.edu/).

## Materials and Methods

### Data Collection and Preprocessing

Raw data files for 11,939 Affymetrix 430 2.0 mouse arrays and 25,229 Affymetrix U133 Plus 2.0 human microarrays were obtained from NIH Gene Expression Omnibus [2]. The data were normalized and probeset expression levels were generated by using the standard robust multichip average algorithm [3].

Next, a threshold was assigned to each probeset using the StepMiner algorithm [4], which was originally designed to fit step functions to time-course data. For this application, the expression values for each probeset were ordered from low-to-high, and StepMiner was used to fit a rising step function to the data that minimizes the difference between the fitted and measured values. This approach places the step at the largest jump from low values to high values (but only if there are sufficiently many expression values on each side of the jump to provide evidence that the jump is not due to noise), and sets the threshold at the point where the step crosses that original data. In the case where the gene expression levels are evenly distributed from low to high, the threshold tends to be near the mean expression level.

### Animals

All animal procedures were approved by the International Animal Care and Use Committee and the Stanford Administrative Panel on Laboratory Animal Care.

### Cell Sorting and Antibodies

All cells were sorted and data collected on a BD FACSAria (Beckton Dickinson, San Jose, CA). FlowJo Software (TreeStar, OR) was used for flow cytometric data analysis. A complete list of all antibodies used in the study is shown in Table 5.

**Table 5:** Clone and Conjugation of Antibodies Used.

Epitope	Clone	Fluorescence	Vendor
AA4.1 (CD93)	AA4.1	APC	eBioscience
B220	RA3-6B2	PE-Cy7,APC-Cy7	eBioscience
B220	RA3-6B2	PacBlue	Weissman lab
CD11b	M1/70	PE-Cy5,PE-Cy7	eBioscience
CD11c	N418	PE-Cy5.5,APC-Cy7	eBioscience
CD122	TM-b1	PE	eBioscinece
CD19	1D3	PE-Cy5.5	eBioscience
CD21	8D9	PE	eBioscience
CD23	B3B4	PECy7	eBioscience
CD25	PC61.5	Pacific orange	Weissman lab
CD27	LG.7F9	APC	eBioscience
CD3	17A2	Pacific Blue	Weissman lab
CD3	17A2	Cy7PE	eBioscience
CD3	2C11	Pacific blue	Weissman lab
CD34	RAM34	FITC	eBioscience
CD4	GK1.5	Alexafluor647,PB	Weissman lab
CD4	GK1.5	PE-Cy7	eBioscience
CD44	IM7	Alexafluor 680	Weissman lab
CD49b	DX5	FITC	eBioscience
CD69	H1.2F3	biotin	eBioscience
CD8a	53.6.7	Alexafluor488	Weissman lab
CD8a	53.6.7	PE-Cy7	eBiosience
c-Kit	2B8	Alexafluor750	eBioscience
FcgrII/III	2.4g2	PacificOrange	Weissman lab
Flk2	A2F10	PE	eBioscience
Gamma-delta TCR	GL3	PE	eBioscience
Gr-1	RB6-8C5	PE-Cy7	eBioscience
IgD	11–26	eFluor 450	eBioscience
IgM	II/41	PECy5	eBioscience
IL7Ra	A7R34	Biotin, PE-Cy5	eBioscience
Ly6d	49H4.3	FITC, A680, PacificOragne	Weissman lab
Mac-1	M1/70	Cy5PE	eBioscience
NK1.1	PK136	PE-Cy5,PE-Cy7	eBioscience
Sca-1	E13-161-7	PacificBlue	Weissman lab
Slamf1	TC15-12F12.2	PE	BioLegend
Thy1.1	OX-7	biotin	Weissman lab
Tie-2	TEK4	biotin	eBioscience
Ter119	TER 119	PE-Cy7	eBioscience
Vcam-1	429	Alexafluor647	BioLegend

### Gene Expression Microarray Analysis

Genome-wide gene expression analysis was performed using Affymetrix GeneChip Mouse Genome 430 2.0 Array (Affymetrix). For each sample, 1 ug of high-quality total RNA was amplified, labeled and hybridized onto the microarray at Stanford PAN facility microarray core according to Affymetrix’s specifications. Microarray data reported in this manuscript is described in accordance with MIAME guidelines. The data has been deposited in GEO public repository (GSE34723).
